# Supplementary material for: Exploratory study of serum protein biomarkers for sudden cardiac arrest using protein extension assay: A case-control study
Source: PLoS One. 2025 Feb 24;20(2):e0319466. doi: 10.1371/journal.pone.0319466 (PMC11849859; doi:10.1371/journal.pone.0319466)
Supplement: S1 Table — (DOCX) [file pone.0319466.s001.docx]

S1 Table. Institutional Review Board (IRB) Numbers of participating hospitals

| Institution | IRB No. | Institution | IRB No. |
| --- | --- | --- | --- |
| Korea University Anam Hospital | 2018AN0148 | Seoul National University Boramae Medical Center | 30-2017-66 |
| Yonsei University Severance Hospital | 4-2017-1201 | Seoul National University Hospital | H-1709-053-883 |
| Korea University Ansan Hospital | 2017AS0729 | Seoul National University Bundang Hospital | B-1711-430-304 |
| Hallym University Kangnam Sacred Heart Hospital | HKS 2018-02-016 | Hallym University Dongtan Sacred Heart Hospital | HDT-2021-04-005-006 |
| Soonchunhyang University Hospital Bucheon | 2018-02-014 | Wonju Severance Christian Hospital | CR317101 |
| Chungbuk National University Hospital | CBNUH-2017-09-009-023 | Kyungpook National University Hospital | KNUH 2017-10-035-013 |
| Chonnam National University Hospital | CNUH-2017-285 | Ulsan University Asan Medical Center | S2021-0855-0006 |
| Sungkyunkwan University Samsung Medical Center | SMC 2018-08-121-015 | Dankook University Hospital | DKUH2018-12-019-012 |
| Dongguk University Ilsan Hospital | DUIH2021-06-001-012) |  |  |

* In compliance with the Declaration of Helsinki, all participants or their proxies provided written informed consent before participating in the study.
