# Supplementary material for: Exploratory study of serum protein biomarkers for sudden cardiac arrest using protein extension assay: A case-control study
Source: PLoS One. 2025 Feb 24;20(2):e0319466. doi: 10.1371/journal.pone.0319466 (PMC11849859; doi:10.1371/journal.pone.0319466)
Supplement: S2 Table — (DOCX) [file pone.0319466.s002.docx]

S2 Table. Full list of proteins in the analysis with protein selection criteria

| No | Abbreviated protein names | Protein names | Panel | UniProt ID | Significant correlation with case^a^ | Weak or no correlation with lactate level^b^ | Weak or no correlation with sampling time^c^ |
| --- | --- | --- | --- | --- | --- | --- | --- |
| 1 | AXL | AXL Receptor Tyrosine Kinase | Cardiometabolic III | P30530 | O | O | O |
| 2 | TIMP4 | TIMP Metallopeptidase Inhibitor 4 | Cardiometabolic III | Q99727 | O | O | O |
| 3 | FGF-23 | Fibroblast Growth Factor 23 | Cardiometabolic II | Q9GZV9 | O | X | O |
| 4 | GT | Gastrotropin | Cardiometabolic II | P51161 | O | X | O |
| 5 | THBS2 | Thrombospondin 2 | Cardiometabolic II | P35442 | O | X | O |
| 6 | CNTN1 | Contactin 1 | Cardiometabolic III | Q12860 | O | X | O |
| 7 | COL1A1 | Collagen Type I Alpha 1 Chain | Cardiometabolic III | P02452 | O | X | O |
| 8 | CTSD | Cathepsin D | Cardiometabolic III | P07339 | O | X | O |
| 9 | ANGPT2 | Angiopoietin 2 | Immuno-oncologic | O15123 | O | X | O |
| 10 | GZMB | Granzyme B | Immuno-oncologic | P10144 | O | X | O |
| 11 | IL10 | Interleukin 10 | Immuno-oncologic | P22301 | O | X | O |
| 12 | LAG3 | Lymphocyte Activating 3 | Immuno-oncologic | P18627 | O | X | O |
| 13 | ADM | Adrenomedullin | Cardiometabolic II | P35318 | O | O | X |
| 14 | CA5A | Carbonic Anhydrase 5A | Cardiometabolic II | P35218 | O | O | X |
| 15 | DCN | Decorin | Cardiometabolic II | P07585 | O | O | X |
| 16 | DECR1 | 2,4-Dienoyl-CoA Reductase 1 | Cardiometabolic II | Q16698 | O | O | X |
| 17 | GH | Growth Hormone 1 | Cardiometabolic II | P01241 | O | O | X |
| 18 | GLO1 | Glyoxalase I | Cardiometabolic II | Q04760 | O | O | X |
| 19 | HAOX1 | HAOX1 | Cardiometabolic II | Q9UJM8 | O | O | X |
| 20 | IGG-FC-RECEPTOR.II.B | Low affinity immunoglobulin gamma Fc region receptor II-b | Cardiometabolic II | P31994 | O | O | X |
| 21 | IL-1RA | Interleukin 1 Receptor Antagonist | Cardiometabolic II | P18510 | O | O | X |
| 22 | IL-4RA | Interleukin 4 Receptor | Cardiometabolic II | P24394 | O | O | X |
| 23 | IL1RL2 | Interleukin 1 Receptor Like 2 | Cardiometabolic II | Q9HB29 | O | O | X |
| 24 | MMP12 | Matrix Metallopeptidase 12 | Cardiometabolic II | P39900 | O | O | X |
| 25 | TGM2 | Transglutaminase 2 | Cardiometabolic II | P21980 | O | O | X |
| 26 | THPO | Thrombopoietin | Cardiometabolic II | P40225 | O | O | X |
| 27 | TNFRSF10A | TNF Receptor Superfamily Member 10a | Cardiometabolic II | O00220 | O | O | X |
| 28 | TNFRSF11A | TNF Receptor Superfamily Member 11a | Cardiometabolic II | Q9Y6Q6 | O | O | X |
| 29 | TRAIL-R2 | TNF Receptor Superfamily Member 10b | Cardiometabolic II | O14763 | O | O | X |
| 30 | BLM-HYDROLASE | Bleomycin Hydrolase | Cardiometabolic III | Q13867 | O | O | X |
| 31 | CSTB | Cystatin B | Cardiometabolic III | P04080 | O | O | X |
| 32 | CXCL16 | C-X-C Motif Chemokine Ligand 16 | Cardiometabolic III | Q9H2A7 | O | O | X |
| 33 | EGFR | Epidermal Growth Factor Receptor | Cardiometabolic III | P00533 | O | O | X |
| 34 | FABP4 | Fatty Acid Binding Protein 4 | Cardiometabolic III | P15090 | O | O | X |
| 35 | GDF-15 | Growth Differentiation Factor 15 | Cardiometabolic III | Q99988 | O | O | X |
| 36 | IGFBP-7 | Insulin Like Growth Factor Binding Protein 7 | Cardiometabolic III | Q16270 | O | O | X |
| 37 | JAM-A | Junctional Adhesion Molecule A | Cardiometabolic III | Q9Y624 | O | O | X |
| 38 | MB | Myoglobin | Cardiometabolic III | P02144 | O | O | X |
| 39 | PI3 | Peptidase Inhibitor 3 | Cardiometabolic III | P19957 | O | O | X |
| 40 | TFF3 | Trefoil Factor 3 | Cardiometabolic III | Q07654 | O | O | X |
| 41 | TNF-R1 | TNF Receptor Superfamily Member 1A | Cardiometabolic III | P19438 | O | O | X |
| 42 | TNF-R2 | TNF Receptor Superfamily Member 1B | Cardiometabolic III | P20333 | O | O | X |
| 43 | TNFRSF10C | TNF Receptor Superfamily Member 10c | Cardiometabolic III | O14798 | O | O | X |
| 44 | TNFRSF14 | TNF Receptor Superfamily Member 14 | Cardiometabolic III | Q92956 | O | O | X |
| 45 | U-PAR | Plasminogen Activator, Urokinase Receptor | Cardiometabolic III | Q03405 | O | O | X |
| 46 | CASP-8 | Caspase 8 | Immuno-oncologic | Q14790 | O | O | X |
| 47 | CD27 | CD27 Molecule | Immuno-oncologic | P26842 | O | O | X |
| 48 | CXCL9 | C-X-C Motif Chemokine Ligand 9 | Immuno-oncologic | Q07325 | O | O | X |
| 49 | GAL-1 | Galectin 1 | Immuno-oncologic | P09382 | O | O | X |
| 50 | GZMH | Granzyme H | Immuno-oncologic | P20718 | O | O | X |
| 51 | IL12RB1 | Interleukin 12 Receptor Subunit Beta 1 | Immuno-oncologic | P42701 | O | O | X |
| 52 | IL7 | Interleukin 7 | Immuno-oncologic | P13232 | O | O | X |
| 53 | LAMP3 | Lysosomal Associated Membrane Protein 3 | Immuno-oncologic | Q9UQV4 | O | O | X |
| 54 | TNFRSF9 | TNF Receptor Superfamily Member 9 | Immuno-oncologic | Q07011 | O | O | X |
| 55 | ACE2 | Angiotensin Converting Enzyme 2 | Cardiometabolic II | Q9BYF1 | O | X | X |
| 56 | CD4 | CD4 Molecule | Cardiometabolic II | P01730 | O | X | X |
| 57 | FABP2 | Fatty Acid Binding Protein 2 | Cardiometabolic II | P12104 | O | X | X |
| 58 | GAL-9 | Galectin 9 | Cardiometabolic II | O00182 | O | X | X |
| 59 | HSP-27 | Heat Shock Protein Family B (Small) Member 1 | Cardiometabolic II | P04792 | O | X | X |
| 60 | IDUA | Alpha-L-Iduronidase | Cardiometabolic II | P35475 | O | X | X |
| 61 | IL-17D | Interleukin 17D | Cardiometabolic II | Q8TAD2 | O | X | X |
| 62 | IL16 | Interleukin 16 | Cardiometabolic II | Q14005 | O | X | X |
| 63 | IL6 | Interleukin 6 | Cardiometabolic II | P05231 | O | X | X |
| 64 | NEMO | Inhibitor Of Nuclear Factor Kappa B Kinase Regulatory Subunit Gamma | Cardiometabolic II | Q9Y6K9 | O | X | X |
| 65 | PAPPA | Pappalysin 1 | Cardiometabolic II | Q13219 | O | X | X |
| 66 | PTX3 | Pentraxin 3 | Cardiometabolic II | P26022 | O | X | X |
| 67 | RAGE | Advanced Glycosylation End-Product Specific Receptor | Cardiometabolic II | Q15109 | O | X | X |
| 68 | STK4 | Serine/Threonine Kinase 4 | Cardiometabolic II | Q13043 | O | X | X |
| 69 | VSIG2 | V-Set And Immunoglobulin Domain Containing 2 | Cardiometabolic II | Q96IQ7 | O | X | X |
| 70 | XCL1 | X-C Motif Chemokine Ligand 1 | Cardiometabolic II | P47992 | O | X | X |
| 71 | CASP-3 | Caspase 3 | Cardiometabolic III | P42574 | O | X | X |
| 72 | CD163 | CD163 Molecule | Cardiometabolic III | Q86VB7 | O | X | X |
| 73 | CPB1 | Carboxypeptidase B1 | Cardiometabolic III | P15086 | O | X | X |
| 74 | EP-CAM | Epithelial Cell Adhesion Molecule | Cardiometabolic III | P16422 | O | X | X |
| 75 | GAL-3 | Galectin 3 | Cardiometabolic III | P17931 | O | X | X |
| 76 | GAL-4 | Galectin 4 | Cardiometabolic III | P56470 | O | X | X |
| 77 | KLK6 | Kallikrein Related Peptidase 6 | Cardiometabolic III | Q92876 | O | X | X |
| 78 | MMP-9 | Matrix Metallopeptidase 9 | Cardiometabolic III | P14780 | O | X | X |
| 79 | PGLYRP1 | Peptidoglycan Recognition Protein 1 | Cardiometabolic III | O75594 | O | X | X |
| 80 | PLC | Phospholipase C | Cardiometabolic III | P98160 | O | X | X |
| 81 | PON3 | Paraoxonase 3 | Cardiometabolic III | Q15166 | O | X | X |
| 82 | RARRES2 | Retinoic Acid Receptor Responder 2 | Cardiometabolic III | Q99969 | O | X | X |
| 83 | RETN | Resistin | Cardiometabolic III | Q9HD89 | O | X | X |
| 84 | SCGB3A2 | Secretoglobin Family 3A Member 2 | Cardiometabolic III | Q96PL1 | O | X | X |
| 85 | T-PA | Plasminogen Activator | Cardiometabolic III | P00750 | O | X | X |
| 86 | VWF | Von Willebrand Factor | Cardiometabolic III | P04275 | O | X | X |
| 87 | ADA | Adenosine Deaminase | Immuno-oncologic | P00813 | O | X | X |
| 88 | ARG1 | Arginase 1 | Immuno-oncologic | P05089 | O | X | X |
| 89 | CAIX | Carbonic Anhydrase 9 | Immuno-oncologic | Q16790 | O | X | X |
| 90 | CD5 | CD5 Molecule | Immuno-oncologic | P06127 | O | X | X |
| 91 | CXCL13 | C-X-C Motif Chemokine Ligand 13 | Immuno-oncologic | O43927 | O | X | X |
| 92 | CXCL5 | C-X-C motif chemokine ligand 12 | Immuno-oncologic | P42830 | O | X | X |
| 93 | FASLG | Fas Ligand | Immuno-oncologic | P48023 | O | X | X |
| 94 | GZMA | Granzyme A | Immuno-oncologic | P12544 | O | X | X |
| 95 | HGF | Hepatocyte Growth Factor | Immuno-oncologic | P14210 | O | X | X |
| 96 | NCR1 | Natural Cytotoxicity Triggering Receptor 1 | Immuno-oncologic | O76036 | O | X | X |
| 97 | NOS3 | Nitric Oxide Synthase 3 | Immuno-oncologic | P29474 | O | X | X |
| 98 | GDF-2 | Growth/differentiation factor 2 | Cardiometabolic II | Q9UK05 | X | O | O |
| 99 | IGFBP-1 | Insulin Like Growth Factor Binding Protein 1 | Cardiometabolic III | P08833 | X | O | O |
| 100 | CCL23 | C-C Motif Chemokine Ligand 23 | Immuno-oncologic | P55773 | X | O | O |
| 101 | ANGPT1 | Angiopoietin-1 | Cardiometabolic II | Q15389 | X | O | O |
| 102 | CXCL1 | C-X-C Motif Chemokine Ligand 1 | Cardiometabolic II | P09341 | X | O | O |
| 103 | SOD2 | Superoxide dismutase 2 | Cardiometabolic II | P04179 | X | O | O |
| 104 | TF | Transferrin | Cardiometabolic II | P13726 | X | O | O |
| 105 | IL-6RA | Interleukin 6 Receptor | Cardiometabolic III | P08887 | X | O | O |
| 106 | IL2-RA | Interleukin 2 Receptor Subunit Alpha | Cardiometabolic III | P01589 | X | O | O |
| 107 | PSP-D | Pulmonary surfactant-associated Protein D | Cardiometabolic III | P35247 | X | O | O |
| 108 | SHPS-1 | Tyrosine-protein phosphatase non-receptor type substrate 1 | Cardiometabolic III | P78324 | X | O | O |
| 109 | TLT-2 | Triggering Receptor Expressed On Myeloid Cells Like 2 | Cardiometabolic III | Q5T2D2 | X | O | O |
| 110 | CD8A | CD8 Subunit Alpha | Immuno-oncologic | P01732 | X | O | O |
| 111 | CX3CL1 | CX3CL1-binding protein 1 | Immuno-oncologic | P78423 | X | O | O |
| 112 | IL12 | Interleukin 12 | Immuno-oncologic | P29459.P29460 | X | O | O |
| 113 | MUC-16 | Mucin 16 | Immuno-oncologic | Q8WXI7 | X | O | O |
| 114 | PDCD1 | Programmed Cell Death 1 | Immuno-oncologic | Q15116 | X | O | O |
| 115 | TNFRSF21 | TNF Receptor Superfamily Member 21 | Immuno-oncologic | O75509 | X | O | O |
| 116 | TRAIL | TNF Superfamily Member 10 | Immuno-oncologic | P50591 | X | O | O |
| 117 | BNP | Natriuretic Peptide B | Cardiometabolic II | P16860 | X | X | O |
| 118 | HO-1 | Heme Oxygenase 1 | Cardiometabolic II | P09601 | X | X | O |
| 119 | SCF | KIT Ligand | Cardiometabolic II | P21583 | X | X | O |
| 120 | SORT1 | Sortilin 1 | Cardiometabolic II | Q99523 | X | X | O |
| 121 | OPG | TNF Receptor Superfamily Member 11b | Cardiometabolic III | O00300 | X | X | O |
| 122 | PRTN3 | Proteinase 3 | Cardiometabolic III | P24158 | X | X | O |
| 123 | ST2 | Suppression Of Tumorigenicity 2 | Cardiometabolic III | Q01638 | X | X | O |
| 124 | ADGRG1 | Adhesion G protein-coupled receptor G1 | Immuno-oncologic | Q9Y653 | X | X | O |
| 125 | CXCL10 | C-X-C motif chemokine ligand 10 | Immuno-oncologic | P02778 | X | X | O |
| 126 | TNFRSF4 | TNF receptor superfamily member 4) | Immuno-oncologic | P43489 | X | X | O |
| 127 | CD84 | CD84 molecule | Cardiometabolic II | Q9UIB8 | X | X | O |
| 128 | LOX-1 | Oxidized Low Density Lipoprotein Receptor 1 | Cardiometabolic II | P78380 | X | X | O |
| 129 | LPL | Lipoprotein lipase | Cardiometabolic II | P06858 | X | X | O |
| 130 | MARCO | Macrophage receptor with collagenous structure | Cardiometabolic II | Q9UEW3 | X | X | O |
| 131 | PD-L2 | Programmed Cell Death 1 Ligand 2 | Cardiometabolic II | Q9BQ51 | X | X | O |
| 132 | CD93 | CD93 molecule | Cardiometabolic III | Q9NPY3 | X | X | O |
| 133 | CDH5 | Cadherin 5 | Cardiometabolic III | P33151 | X | X | O |
| 134 | CTSZ | Cathepsin Z | Cardiometabolic III | Q9UBR2 | X | X | O |
| 135 | ICAM-2 | Intercellular Adhesion Molecule 2 | Cardiometabolic III | P13598 | X | X | O |
| 136 | LDL-RECEPTOR | Low Density Lipoprotein Receptor | Cardiometabolic III | P01130 | X | X | O |
| 137 | MEPE | Matrix extracellular phosphoglycoprotein | Cardiometabolic III | Q9NQ76 | X | X | O |
| 138 | MMP-3 | Matrix Metallopeptidase 3 | Cardiometabolic III | P08254 | X | X | O |
| 139 | MPO | Myeloperoxidase | Cardiometabolic III | P05164 | X | X | O |
| 140 | OPN | Secreted Phosphoprotein 1 | Cardiometabolic III | P10451 | X | X | O |
| 141 | TNFSF13B | TNF superfamily member 13b | Cardiometabolic III | Q9Y275 | X | X | O |
| 142 | TR-AP | Acid Phosphatase 5, Tartrate Resistant | Cardiometabolic III | P13686 | X | X | O |
| 143 | CD70 | CD70 molecule | Immuno-oncologic | P32970 | X | X | O |
| 144 | CRTAM | Cytotoxic and regulatory T cell molecule | Immuno-oncologic | O95727 | X | X | O |
| 145 | IL8 | C-X-C Motif Chemokine Ligand 8 | Immuno-oncologic | P10145 | X | X | O |
| 146 | KIR3DL1 | Killer cell immunoglobulin like receptor, three Ig domains and long cytoplasmic tail 1 | Immuno-oncologic | P43629 | X | X | O |
| 147 | MCP-3 | C-C Motif Chemokine Ligand 7 | Immuno-oncologic | P80098 | X | X | O |
| 148 | TNFRSF12A | TNF receptor superfamily member 12A | Immuno-oncologic | Q9NP84 | X | X | O |
| 149 | TNFSF14 | TNF superfamily member 14 | Immuno-oncologic | O43557 | X | X | O |
| 150 | TWEAK | TNF Superfamily Member 12 | Immuno-oncologic | O43508 | X | X | O |
| 151 | MMP7 | Matrix Metallopeptidase 7 | Cardiometabolic II | P09237 | X | O | X |
| 152 | EPHB4 | EPH Receptor B4 | Cardiometabolic III | P54760 | X | O | X |
| 153 | SPON1 | Spondin 1 | Cardiometabolic III | Q9HCB6 | X | O | X |
| 154 | CD40 | CD40 Molecule | Immuno-oncologic | P25942 | X | O | X |
| 155 | CSF-1 | Colony Stimulating Factor 1 | Immuno-oncologic | P09603 | X | O | X |
| 156 | ICOSLG | Inducible T Cell Costimulator Ligand | Immuno-oncologic | O75144 | X | O | X |
| 157 | PD-L1 | CD274 Molecule | Immuno-oncologic | Q9NZQ7 | X | O | X |
| 158 | VEGFR-2 | Kinase Insert Domain Receptor | Immuno-oncologic | P35968 | X | O | X |
| 159 | AGRP | Agouti Related Neuropeptide | Cardiometabolic II | O00253 | X | O | X |
| 160 | BMP-6 | Bone Morphogenetic Protein 6 | Cardiometabolic II | P22004 | X | O | X |
| 161 | BOC | Brother Of CDO | Cardiometabolic II | Q9BWV1 | X | O | X |
| 162 | CCL3 | C-C Motif Chemokine Ligand 3 | Cardiometabolic II | P10147 | X | O | X |
| 163 | CTRC | Chymotrypsin C | Cardiometabolic II | Q99895 | X | O | X |
| 164 | CTSL1 | Cathepsin L1 | Cardiometabolic II | P07711 | X | O | X |
| 165 | DKK-1 | Dickkopf WNT Signaling Pathway Inhibitor 1 | Cardiometabolic II | O94907 | X | O | X |
| 166 | PAR-1 | Coagulation Factor II Thrombin Receptor | Cardiometabolic II | P25116 | X | O | X |
| 167 | PGF | Placental Growth Factor | Cardiometabolic II | P49763 | X | O | X |
| 168 | PIGR | Polymeric Immunoglobulin Receptor | Cardiometabolic II | P01833 | X | O | X |
| 169 | PRELP | Proline And Arginine Rich End Leucine Rich Repeat Protein | Cardiometabolic II | P51888 | X | O | X |
| 170 | PRSS27 | Serine Protease 27 | Cardiometabolic II | Q9BQR3 | X | O | X |
| 171 | SPON2 | Spondin 2 | Cardiometabolic II | Q9BUD6 | X | O | X |
| 172 | ALCAM | Activated Leukocyte Cell Adhesion Molecule | Cardiometabolic III | Q13740 | X | O | X |
| 173 | AP-N | Alanyl Aminopeptidase, Membrane | Cardiometabolic III | P15144 | X | O | X |
| 174 | CCL15 | C-C Motif Chemokine Ligand 15 | Cardiometabolic III | Q16663 | X | O | X |
| 175 | DLK-1 | Delta Like Non-Canonical Notch Ligand 1 | Cardiometabolic III | P80370 | X | O | X |
| 176 | FAS | Fas Cell Surface Death Receptor | Cardiometabolic III | P25445 | X | O | X |
| 177 | GP6 | Glycoprotein VI Platelet | Cardiometabolic III | Q9HCN6 | X | O | X |
| 178 | GRN | Granulin Precursor | Cardiometabolic III | P28799 | X | O | X |
| 179 | IL-17RA | Interleukin 17 Receptor A | Cardiometabolic III | Q96F46 | X | O | X |
| 180 | IL-18BP | Interleukin 18 Binding Protein | Cardiometabolic III | O95998 | X | O | X |
| 181 | IL-1RT1 | Interleukin 1 Receptor Type 1 | Cardiometabolic III | P14778 | X | O | X |
| 182 | ITGB2 | Integrin Subunit Beta 2 | Cardiometabolic III | P05107 | X | O | X |
| 183 | LTBR | Lymphotoxin Beta Receptor | Cardiometabolic III | P36941 | X | O | X |
| 184 | MCP-1 | C-C Motif Chemokine Ligand 2 | Cardiometabolic III | P13500 | X | O | X |
| 185 | NOTCH-3 | Notch Receptor 3 | Cardiometabolic III | Q9UM47 | X | O | X |
| 186 | PCSK9 | Proprotein Convertase Subtilisin/Kexin Type 9 | Cardiometabolic III | Q8NBP7 | X | O | X |
| 187 | PDGF-SUBUNIT-A | Platelet Derived Growth Factor Subunit B | Cardiometabolic III | P04085 | X | O | X |
| 188 | SELP | Selectin P | Cardiometabolic III | P16109 | X | O | X |
| 189 | TFPI | Tissue Factor Pathway Inhibitor | Cardiometabolic III | P10646 | X | O | X |
| 190 | TR | Transferrin receptor protein 1 | Cardiometabolic III | P02786 | X | O | X |
| 191 | CCL19 | C-C Motif Chemokine Ligand 19 | Immuno-oncologic | Q99731 | X | O | X |
| 192 | CCL4 | C-C Motif Chemokine Ligand 4 | Immuno-oncologic | P13236 | X | O | X |
| 193 | CD244 | CD244 Molecule | Immuno-oncologic | Q9BZW8 | X | O | X |
| 194 | EGF | Epidermal Growth Factor | Immuno-oncologic | P01133 | X | O | X |
| 195 | FGF2 | Fibroblast Growth Factor 2 | Immuno-oncologic | P09038 | X | O | X |
| 196 | KLRD1 | Killer Cell Lectin Like Receptor D1 | Immuno-oncologic | Q13241 | X | O | X |
| 197 | LAP-TGF-BETA.1 | Transforming Growth Factor Beta 1 | Immuno-oncologic | P01137 | X | O | X |
| 198 | MCP-4 | C-C Motif Chemokine Ligand 13 | Immuno-oncologic | Q99616 | X | O | X |
| 199 | TNF | Tumor Necrosis Factor | Immuno-oncologic | P01375 | X | O | X |
| 200 | VEGFA | Vascular Endothelial Growth Factor A | Immuno-oncologic | P15692 | X | O | X |
| 201 | ADAM-TS13 | ADAM Metallopeptidase With Thrombospondin Type 1 Motif 13 | Cardiometabolic II | Q76LX8 | X | X | X |
| 202 | CEACAM8 | CEA Cell Adhesion Molecule 8 | Cardiometabolic II | P31997 | X | X | X |
| 203 | FS | Follistatin | Cardiometabolic II | P19883 | X | X | X |
| 204 | IL18 | Interleukin 18 | Cardiometabolic II | Q14116 | X | X | X |
| 205 | TIE2 | TEK Receptor Tyrosine Kinase | Cardiometabolic II | Q02763 | X | X | X |
| 206 | TNFRSF13B | TNF Receptor Superfamily Member 13B | Cardiometabolic II | O14836 | X | X | X |
| 207 | CHI3L1 | Chitinase 3 Like 1 | Cardiometabolic III | P36222 | X | X | X |
| 208 | CPA1 | Carboxypeptidase A1 | Cardiometabolic III | P15085 | X | X | X |
| 209 | UPA | Plasminogen Activator, Urokinase | Cardiometabolic III | P00749 | X | X | X |
| 210 | CCL20 | C-C motif chemokine ligand 20 | Immuno-oncologic | P78556 | X | X | X |
| 211 | AMBP | Alpha-1-microglobulin/bikunin precursor | Cardiometabolic II | P02760 | X | X | X |
| 212 | CCL17 | C-C motif chemokine ligand 17 | Cardiometabolic II | Q92583 | X | X | X |
| 213 | CD40-L | CD40 Ligand | Cardiometabolic II | P29965 | X | X | X |
| 214 | FGF-21 | Fibroblast Growth Factor 21 | Cardiometabolic II | Q9NSA1 | X | X | X |
| 215 | GIF | Cobalamin Binding Intrinsic Factor | Cardiometabolic II | P27352 | X | X | X |
| 216 | HB-EGF | Heparin Binding EGF Like Growth Factor | Cardiometabolic II | Q99075 | X | X | X |
| 217 | HOSCAR | Osteoclast Associated Ig-Like Receptor | Cardiometabolic II | Q8IYS5 | X | X | X |
| 218 | IL-27 | Interleukin 27 | Cardiometabolic II | Q8NEV9/Q14213 | X | X | X |
| 219 | ITGB1BP2 | Integrin subunit beta 1 binding protein 2 | Cardiometabolic II | Q9UKP3 | X | X | X |
| 220 | KIM1 | Hepatitis A Virus Cellular Receptor 1 | Cardiometabolic II | Q96D42 | X | X | X |
| 221 | LEP | Leptin | Cardiometabolic II | P41159 | X | X | X |
| 222 | MERTK | MER proto-oncogene, tyrosine kinase | Cardiometabolic II | Q12866 | X | X | X |
| 223 | PDGF-SUBUNIT-B | Platelet Derived Growth Factor Subunit A | Cardiometabolic II | P01127 | X | X | X |
| 224 | PRSS8 | Serine Protease 8 | Cardiometabolic II | Q16651 | X | X | X |
| 225 | PSGL-1 | Selectin P Ligand | Cardiometabolic II | Q14242 | X | X | X |
| 226 | REN | Renin | Cardiometabolic II | P00797 | X | X | X |
| 227 | SERPINA12 | Serpin family A member 12 | Cardiometabolic II | Q8IW75 | X | X | X |
| 228 | SLAMF7 | SLAM family member 7 | Cardiometabolic II | Q9NQ25 | X | X | X |
| 229 | SRC | SRC proto-oncogene, non-receptor tyrosine kinase | Cardiometabolic II | P12931 | X | X | X |
| 230 | TM | Thrombomodulin | Cardiometabolic II | P07204 | X | X | X |
| 231 | VEGFD | Vascular endothelial growth factor D | Cardiometabolic II | O43915 | X | X | X |
| 232 | AZU1 | Azurocidin 1 | Cardiometabolic III | P20160 | X | X | X |
| 233 | CCL16 | C-C motif chemokine ligand 16 | Cardiometabolic III | O15467 | X | X | X |
| 234 | CCL24 | C-C motif chemokine ligand 24 | Cardiometabolic III | O00175 | X | X | X |
| 235 | IGFBP-2 | Insulin Like Growth Factor Binding Protein 2 | Cardiometabolic III | P18065 | X | X | X |
| 236 | IL-1RT2 | Interleukin 1 Receptor Type 2 | Cardiometabolic III | P27930 | X | X | X |
| 237 | MMP-2 | Matrix Metallopeptidase 2 | Cardiometabolic III | P08253 | X | X | X |
| 238 | PAI | Serpin Family E Member 1 | Cardiometabolic III | P05121 | X | X | X |
| 239 | PECAM-1 | Platelet And Endothelial Cell Adhesion Molecule 1 | Cardiometabolic III | P16284 | X | X | X |
| 240 | SELE | Selectin E | Cardiometabolic III | P16581 | X | X | X |
| 241 | CD83 | CD83 molecule | Immuno-oncologic | Q01151 | X | X | X |
| 242 | CXCL11 | C-X-C motif chemokine ligand 11 | Immuno-oncologic | O14625 | X | X | X |
| 243 | IFN-GAMMA | Interferon Gamma | Immuno-oncologic | P01579 | X | X | X |
| 244 | IL15 | Interleukin 15 | Immuno-oncologic | P40933 | X | X | X |
| 245 | MCP-2 | C-C Motif Chemokine Ligand 8 | Immuno-oncologic | P80075 | X | X | X |
| 246 | MIC-A-B | MHC Class I Polypeptide-Related Sequence A, B | Immuno-oncologic | Q29983/Q29980 | X | X | X |

^a^Strong correlation with case (label=1 for case and label=0 for control) (|Spearman correlation coefficient|>0.6)

^b^Weak or no correlation with lactate level (|Spearman correlation coefficient|<0.1)

^c^Weak or no correlation with arrest onset-to-sampling time (|Spearman correlation coefficient|<0.1)
