# Supplementary material for: Exploratory study of serum protein biomarkers for sudden cardiac arrest using protein extension assay: A case-control study
Source: PLoS One. 2025 Feb 24;20(2):e0319466. doi: 10.1371/journal.pone.0319466 (PMC11849859; doi:10.1371/journal.pone.0319466)
Supplement: S3 Table — (DOCX) [file pone.0319466.s003.docx]

S3 Table. Full list of proteins in the analysis with correlation coefficients at each criteria

| No | Abbreviated protein names | Protein names | Panel | UniProt ID | Correlation coefficient with case | Correlation coefficient with lactate | Correlation coefficient with sampling time |
| --- | --- | --- | --- | --- | --- | --- | --- |
| 1 | AXL | AXL Receptor Tyrosine Kinase | Cardiometabolic III | P30530 | 0.681 | -0.047 | -0.035 |
| 2 | TIMP4 | TIMP Metallopeptidase Inhibitor 4 | Cardiometabolic III | Q99727 | 0.635 | 0.034 | 0.090 |
| 3 | FGF-23 | Fibroblast Growth Factor 23 | Cardiometabolic II | Q9GZV9 | 0.543 | 0.105 | 0.010 |
| 4 | GT | Gastrotropin | Cardiometabolic II | P51161 | 0.740 | -0.235 | 0.076 |
| 5 | THBS2 | Thrombospondin 2 | Cardiometabolic II | P35442 | 0.562 | 0.144 | 0.069 |
| 6 | CNTN1 | Contactin 1 | Cardiometabolic III | Q12860 | -0.530 | -0.161 | -0.072 |
| 7 | COL1A1 | Collagen Type I Alpha 1 Chain | Cardiometabolic III | P02452 | 0.599 | 0.258 | 0.060 |
| 8 | CTSD | Cathepsin D | Cardiometabolic III | P07339 | 0.546 | 0.268 | 0.069 |
| 9 | ANGPT2 | Angiopoietin 2 | Immuno-oncologic | O15123 | 0.833 | 0.145 | -0.002 |
| 10 | GZMB | Granzyme B | Immuno-oncologic | P10144 | 0.665 | 0.101 | 0.061 |
| 11 | IL10 | Interleukin 10 | Immuno-oncologic | P22301 | 0.649 | 0.102 | 0.000 |
| 12 | LAG3 | Lymphocyte Activating 3 | Immuno-oncologic | P18627 | 0.623 | 0.439 | -0.026 |
| 13 | ADM | Adrenomedullin | Cardiometabolic II | P35318 | -0.568 | 0.016 | -0.244 |
| 14 | CA5A | Carbonic Anhydrase 5A | Cardiometabolic II | P35218 | 0.839 | 0.022 | 0.421 |
| 15 | DCN | Decorin | Cardiometabolic II | P07585 | 0.633 | -0.098 | 0.275 |
| 16 | DECR1 | 2,4-Dienoyl-CoA Reductase 1 | Cardiometabolic II | Q16698 | 0.843 | 0.083 | 0.518 |
| 17 | GH | Growth Hormone 1 | Cardiometabolic II | P01241 | 0.839 | -0.037 | 0.263 |
| 18 | GLO1 | Glyoxalase I | Cardiometabolic II | Q04760 | 0.824 | 0.089 | 0.477 |
| 19 | HAOX1 | HAOX1 | Cardiometabolic II | Q9UJM8 | 0.787 | -0.013 | 0.365 |
| 20 | IGG-FC-RECEPTOR.II.B | Low affinity immunoglobulin gamma Fc region receptor II-b | Cardiometabolic II | P31994 | 0.680 | 0.084 | 0.155 |
| 21 | IL-1RA | Interleukin 1 Receptor Antagonist | Cardiometabolic II | P18510 | 0.684 | 0.044 | 0.195 |
| 22 | IL-4RA | Interleukin 4 Receptor | Cardiometabolic II | P24394 | 0.692 | -0.012 | 0.362 |
| 23 | IL1RL2 | Interleukin 1 Receptor Like 2 | Cardiometabolic II | Q9HB29 | 0.649 | 0.032 | 0.208 |
| 24 | MMP12 | Matrix Metallopeptidase 12 | Cardiometabolic II | P39900 | 0.570 | 0.075 | 0.394 |
| 25 | TGM2 | Transglutaminase 2 | Cardiometabolic II | P21980 | 0.724 | 0.081 | 0.269 |
| 26 | THPO | Thrombopoietin | Cardiometabolic II | P40225 | -0.562 | 0.009 | 0.188 |
| 27 | TNFRSF10A | TNF Receptor Superfamily Member 10a | Cardiometabolic II | O00220 | 0.622 | 0.082 | 0.294 |
| 28 | TNFRSF11A | TNF Receptor Superfamily Member 11a | Cardiometabolic II | Q9Y6Q6 | 0.814 | 0.056 | 0.388 |
| 29 | TRAIL-R2 | TNF Receptor Superfamily Member 10b | Cardiometabolic II | O14763 | 0.793 | 0.094 | 0.356 |
| 30 | BLM-HYDROLASE | Bleomycin Hydrolase | Cardiometabolic III | Q13867 | 0.617 | 0.049 | 0.297 |
| 31 | CSTB | Cystatin B | Cardiometabolic III | P04080 | 0.866 | 0.084 | 0.494 |
| 32 | CXCL16 | C-X-C Motif Chemokine Ligand 16 | Cardiometabolic III | Q9H2A7 | 0.662 | 0.035 | 0.163 |
| 33 | EGFR | Epidermal Growth Factor Receptor | Cardiometabolic III | P00533 | -0.656 | 0.032 | -0.147 |
| 34 | FABP4 | Fatty Acid Binding Protein 4 | Cardiometabolic III | P15090 | 0.855 | 0.059 | 0.378 |
| 35 | GDF-15 | Growth Differentiation Factor 15 | Cardiometabolic III | Q99988 | 0.702 | 0.079 | 0.111 |
| 36 | IGFBP-7 | Insulin Like Growth Factor Binding Protein 7 | Cardiometabolic III | Q16270 | 0.517 | 0.041 | 0.163 |
| 37 | JAM-A | Junctional Adhesion Molecule A | Cardiometabolic III | Q9Y624 | 0.850 | 0.030 | 0.525 |
| 38 | MB | Myoglobin | Cardiometabolic III | P02144 | 0.850 | -0.021 | 0.128 |
| 39 | PI3 | Peptidase Inhibitor 3 | Cardiometabolic III | P19957 | 0.549 | -0.020 | 0.132 |
| 40 | TFF3 | Trefoil Factor 3 | Cardiometabolic III | Q07654 | 0.822 | -0.021 | 0.251 |
| 41 | TNF-R1 | TNF Receptor Superfamily Member 1A | Cardiometabolic III | P19438 | 0.866 | -0.019 | 0.555 |
| 42 | TNF-R2 | TNF Receptor Superfamily Member 1B | Cardiometabolic III | P20333 | 0.816 | 0.089 | 0.273 |
| 43 | TNFRSF10C | TNF Receptor Superfamily Member 10c | Cardiometabolic III | O14798 | 0.531 | -0.049 | 0.175 |
| 44 | TNFRSF14 | TNF Receptor Superfamily Member 14 | Cardiometabolic III | Q92956 | 0.752 | -0.017 | 0.481 |
| 45 | U-PAR | Plasminogen Activator, Urokinase Receptor | Cardiometabolic III | Q03405 | 0.746 | -0.001 | 0.469 |
| 46 | CASP-8 | Caspase 8 | Immuno-oncologic | Q14790 | 0.689 | 0.099 | 0.349 |
| 47 | CD27 | CD27 Molecule | Immuno-oncologic | P26842 | 0.567 | 0.081 | 0.235 |
| 48 | CXCL9 | C-X-C Motif Chemokine Ligand 9 | Immuno-oncologic | Q07325 | 0.750 | -0.026 | 0.257 |
| 49 | GAL-1 | Galectin 1 | Immuno-oncologic | P09382 | 0.839 | 0.026 | 0.104 |
| 50 | GZMH | Granzyme H | Immuno-oncologic | P20718 | 0.785 | 0.078 | 0.135 |
| 51 | IL12RB1 | Interleukin 12 Receptor Subunit Beta 1 | Immuno-oncologic | P42701 | 0.542 | 0.046 | 0.395 |
| 52 | IL7 | Interleukin 7 | Immuno-oncologic | P13232 | -0.688 | -0.045 | -0.288 |
| 53 | LAMP3 | Lysosomal Associated Membrane Protein 3 | Immuno-oncologic | Q9UQV4 | 0.540 | -0.089 | 0.451 |
| 54 | TNFRSF9 | TNF Receptor Superfamily Member 9 | Immuno-oncologic | Q07011 | 0.581 | 0.011 | 0.113 |
| 55 | ACE2 | Angiotensin Converting Enzyme 2 | Cardiometabolic II | Q9BYF1 | 0.590 | 0.282 | 0.179 |
| 56 | CD4 | CD4 Molecule | Cardiometabolic II | P01730 | 0.862 | 0.205 | 0.347 |
| 57 | FABP2 | Fatty Acid Binding Protein 2 | Cardiometabolic II | P12104 | 0.811 | -0.284 | 0.164 |
| 58 | GAL-9 | Galectin 9 | Cardiometabolic II | O00182 | 0.771 | 0.143 | 0.331 |
| 59 | HSP-27 | Heat Shock Protein Family B (Small) Member 1 | Cardiometabolic II | P04792 | 0.827 | -0.216 | 0.425 |
| 60 | IDUA | Alpha-L-Iduronidase | Cardiometabolic II | P35475 | 0.742 | 0.344 | -0.254 |
| 61 | IL-17D | Interleukin 17D | Cardiometabolic II | Q8TAD2 | 0.571 | 0.109 | 0.115 |
| 62 | IL16 | Interleukin 16 | Cardiometabolic II | Q14005 | 0.777 | 0.186 | 0.300 |
| 63 | IL6 | Interleukin 6 | Cardiometabolic II | P05231 | 0.759 | 0.279 | 0.269 |
| 64 | NEMO | Inhibitor Of Nuclear Factor Kappa B Kinase Regulatory Subunit Gamma | Cardiometabolic II | Q9Y6K9 | 0.724 | 0.257 | 0.194 |
| 65 | PAPPA | Pappalysin 1 | Cardiometabolic II | Q13219 | 0.748 | 0.174 | 0.350 |
| 66 | PTX3 | Pentraxin 3 | Cardiometabolic II | P26022 | 0.635 | 0.266 | 0.217 |
| 67 | RAGE | Advanced Glycosylation End-Product Specific Receptor | Cardiometabolic II | Q15109 | 0.843 | -0.124 | -0.133 |
| 68 | STK4 | Serine/Threonine Kinase 4 | Cardiometabolic II | Q13043 | 0.525 | 0.248 | 0.122 |
| 69 | VSIG2 | V-Set And Immunoglobulin Domain Containing 2 | Cardiometabolic II | Q96IQ7 | 0.777 | 0.173 | 0.206 |
| 70 | XCL1 | X-C Motif Chemokine Ligand 1 | Cardiometabolic II | P47992 | 0.531 | 0.305 | 0.484 |
| 71 | CASP-3 | Caspase 3 | Cardiometabolic III | P42574 | 0.821 | 0.243 | 0.347 |
| 72 | CD163 | CD163 Molecule | Cardiometabolic III | Q86VB7 | 0.626 | 0.169 | -0.522 |
| 73 | CPB1 | Carboxypeptidase B1 | Cardiometabolic III | P15086 | 0.617 | 0.336 | 0.358 |
| 74 | EP-CAM | Epithelial Cell Adhesion Molecule | Cardiometabolic III | P16422 | 0.658 | 0.270 | 0.138 |
| 75 | GAL-3 | Galectin 3 | Cardiometabolic III | P17931 | 0.786 | 0.126 | 0.287 |
| 76 | GAL-4 | Galectin 4 | Cardiometabolic III | P56470 | 0.866 | 0.147 | 0.305 |
| 77 | KLK6 | Kallikrein Related Peptidase 6 | Cardiometabolic III | Q92876 | 0.693 | -0.307 | 0.300 |
| 78 | MMP-9 | Matrix Metallopeptidase 9 | Cardiometabolic III | P14780 | 0.521 | 0.172 | 0.257 |
| 79 | PGLYRP1 | Peptidoglycan Recognition Protein 1 | Cardiometabolic III | O75594 | 0.743 | 0.165 | 0.182 |
| 80 | PLC | Phospholipase C | Cardiometabolic III | P98160 | 0.528 | 0.184 | 0.132 |
| 81 | PON3 | Paraoxonase 3 | Cardiometabolic III | Q15166 | -0.543 | -0.251 | -0.239 |
| 82 | RARRES2 | Retinoic Acid Receptor Responder 2 | Cardiometabolic III | Q99969 | -0.662 | -0.145 | -0.191 |
| 83 | RETN | Resistin | Cardiometabolic III | Q9HD89 | 0.573 | 0.108 | 0.170 |
| 84 | SCGB3A2 | Secretoglobin Family 3A Member 2 | Cardiometabolic III | Q96PL1 | 0.716 | -0.337 | 0.589 |
| 85 | T-PA | Plasminogen Activator | Cardiometabolic III | P00750 | 0.804 | 0.329 | 0.107 |
| 86 | VWF | Von Willebrand Factor | Cardiometabolic III | P04275 | 0.866 | 0.160 | 0.184 |
| 87 | ADA | Adenosine Deaminase | Immuno-oncologic | P00813 | 0.828 | 0.373 | 0.193 |
| 88 | ARG1 | Arginase 1 | Immuno-oncologic | P05089 | 0.834 | -0.147 | 0.336 |
| 89 | CAIX | Carbonic Anhydrase 9 | Immuno-oncologic | Q16790 | 0.598 | 0.270 | 0.265 |
| 90 | CD5 | CD5 Molecule | Immuno-oncologic | P06127 | 0.654 | 0.147 | 0.307 |
| 91 | CXCL13 | C-X-C Motif Chemokine Ligand 13 | Immuno-oncologic | O43927 | 0.797 | 0.189 | 0.266 |
| 92 | CXCL5 | C-X-C motif chemokine ligand 12 | Immuno-oncologic | P42830 | -0.523 | 0.418 | -0.247 |
| 93 | FASLG | Fas Ligand | Immuno-oncologic | P48023 | 0.632 | 0.252 | 0.180 |
| 94 | GZMA | Granzyme A | Immuno-oncologic | P12544 | 0.767 | 0.193 | 0.463 |
| 95 | HGF | Hepatocyte Growth Factor | Immuno-oncologic | P14210 | 0.762 | 0.177 | 0.390 |
| 96 | NCR1 | Natural Cytotoxicity Triggering Receptor 1 | Immuno-oncologic | O76036 | 0.790 | 0.167 | 0.468 |
| 97 | NOS3 | Nitric Oxide Synthase 3 | Immuno-oncologic | P29474 | 0.812 | 0.224 | 0.198 |
| 98 | GDF-2 | Growth/differentiation factor 2 | Cardiometabolic II | Q9UK05 | -0.480 | 0.094 | 0.099 |
| 99 | IGFBP-1 | Insulin Like Growth Factor Binding Protein 1 | Cardiometabolic III | P08833 | 0.403 | 0.072 | 0.076 |
| 100 | CCL23 | C-C Motif Chemokine Ligand 23 | Immuno-oncologic | P55773 | 0.473 | -0.088 | 0.083 |
| 101 | ANGPT1 | Angiopoietin-1 | Cardiometabolic II | Q15389 | -0.211 | 0.006 | -0.017 |
| 102 | CXCL1 | C-X-C Motif Chemokine Ligand 1 | Cardiometabolic II | P09341 | 0.078 | 0.091 | 0.087 |
| 103 | SOD2 | Superoxide dismutase 2 | Cardiometabolic II | P04179 | -0.235 | 0.015 | 0.029 |
| 104 | TF | Transferrin | Cardiometabolic II | P13726 | 0.048 | 0.084 | 0.098 |
| 105 | IL-6RA | Interleukin 6 Receptor | Cardiometabolic III | P08887 | 0.053 | 0.014 | -0.036 |
| 106 | IL2-RA | Interleukin 2 Receptor Subunit Alpha | Cardiometabolic III | P01589 | 0.259 | 0.001 | 0.021 |
| 107 | PSP-D | Pulmonary surfactant-associated Protein D | Cardiometabolic III | P35247 | -0.002 | -0.057 | 0.010 |
| 108 | SHPS-1 | Tyrosine-protein phosphatase non-receptor type substrate 1 | Cardiometabolic III | P78324 | 0.373 | -0.069 | -0.036 |
| 109 | TLT-2 | Triggering Receptor Expressed On Myeloid Cells Like 2 | Cardiometabolic III | Q5T2D2 | -0.042 | 0.050 | 0.051 |
| 110 | CD8A | CD8 Subunit Alpha | Immuno-oncologic | P01732 | -0.408 | 0.046 | -0.098 |
| 111 | CX3CL1 | CX3CL1-binding protein 1 | Immuno-oncologic | P78423 | 0.227 | -0.021 | 0.066 |
| 112 | IL12 | Interleukin 12 | Immuno-oncologic | P29459.P29460 | 0.060 | -0.039 | 0.045 |
| 113 | MUC-16 | Mucin 16 | Immuno-oncologic | Q8WXI7 | 0.169 | -0.048 | -0.005 |
| 114 | PDCD1 | Programmed Cell Death 1 | Immuno-oncologic | Q15116 | 0.028 | -0.095 | 0.054 |
| 115 | TNFRSF21 | TNF Receptor Superfamily Member 21 | Immuno-oncologic | O75509 | 0.302 | -0.072 | -0.084 |
| 116 | TRAIL | TNF Superfamily Member 10 | Immuno-oncologic | P50591 | 0.185 | -0.050 | 0.032 |
| 117 | BNP | Natriuretic Peptide B | Cardiometabolic II | P16860 | 0.499 | -0.169 | -0.086 |
| 118 | HO-1 | Heme Oxygenase 1 | Cardiometabolic II | P09601 | 0.471 | -0.130 | 0.049 |
| 119 | SCF | KIT Ligand | Cardiometabolic II | P21583 | -0.514 | -0.233 | 0.076 |
| 120 | SORT1 | Sortilin 1 | Cardiometabolic II | Q99523 | -0.416 | 0.293 | 0.026 |
| 121 | OPG | TNF Receptor Superfamily Member 11b | Cardiometabolic III | O00300 | 0.493 | 0.151 | 0.049 |
| 122 | PRTN3 | Proteinase 3 | Cardiometabolic III | P24158 | 0.468 | 0.249 | -0.075 |
| 123 | ST2 | Suppression Of Tumorigenicity 2 | Cardiometabolic III | Q01638 | 0.444 | 0.211 | -0.023 |
| 124 | ADGRG1 | Adhesion G protein-coupled receptor G1 | Immuno-oncologic | Q9Y653 | 0.468 | 0.197 | 0.008 |
| 125 | CXCL10 | C-X-C motif chemokine ligand 10 | Immuno-oncologic | P02778 | 0.440 | 0.392 | 0.018 |
| 126 | TNFRSF4 | TNF receptor superfamily member 4) | Immuno-oncologic | P43489 | 0.408 | -0.139 | -0.016 |
| 127 | CD84 | CD84 molecule | Cardiometabolic II | Q9UIB8 | -0.384 | 0.180 | -0.015 |
| 128 | LOX-1 | Oxidized Low Density Lipoprotein Receptor 1 | Cardiometabolic II | P78380 | 0.402 | 0.348 | -0.030 |
| 129 | LPL | Lipoprotein lipase | Cardiometabolic II | P06858 | -0.124 | -0.161 | -0.060 |
| 130 | MARCO | Macrophage receptor with collagenous structure | Cardiometabolic II | Q9UEW3 | -0.071 | -0.128 | -0.026 |
| 131 | PD-L2 | Programmed Cell Death 1 Ligand 2 | Cardiometabolic II | Q9BQ51 | 0.306 | 0.101 | 0.042 |
| 132 | CD93 | CD93 molecule | Cardiometabolic III | Q9NPY3 | 0.201 | -0.130 | 0.038 |
| 133 | CDH5 | Cadherin 5 | Cardiometabolic III | P33151 | -0.268 | -0.102 | -0.081 |
| 134 | CTSZ | Cathepsin Z | Cardiometabolic III | Q9UBR2 | 0.364 | -0.115 | 0.080 |
| 135 | ICAM-2 | Intercellular Adhesion Molecule 2 | Cardiometabolic III | P13598 | 0.223 | 0.243 | 0.098 |
| 136 | LDL-RECEPTOR | Low Density Lipoprotein Receptor | Cardiometabolic III | P01130 | 0.011 | 0.178 | 0.020 |
| 137 | MEPE | Matrix extracellular phosphoglycoprotein | Cardiometabolic III | Q9NQ76 | -0.119 | -0.165 | -0.088 |
| 138 | MMP-3 | Matrix Metallopeptidase 3 | Cardiometabolic III | P08254 | -0.008 | -0.300 | 0.090 |
| 139 | MPO | Myeloperoxidase | Cardiometabolic III | P05164 | 0.251 | 0.219 | -0.004 |
| 140 | OPN | Secreted Phosphoprotein 1 | Cardiometabolic III | P10451 | -0.061 | -0.188 | -0.058 |
| 141 | TNFSF13B | TNF superfamily member 13b | Cardiometabolic III | Q9Y275 | 0.084 | 0.164 | 0.092 |
| 142 | TR-AP | Acid Phosphatase 5, Tartrate Resistant | Cardiometabolic III | P13686 | 0.031 | -0.269 | 0.085 |
| 143 | CD70 | CD70 molecule | Immuno-oncologic | P32970 | -0.111 | -0.141 | -0.099 |
| 144 | CRTAM | Cytotoxic and regulatory T cell molecule | Immuno-oncologic | O95727 | 0.043 | -0.141 | -0.080 |
| 145 | IL8 | C-X-C Motif Chemokine Ligand 8 | Immuno-oncologic | P10145 | -0.176 | 0.149 | 0.035 |
| 146 | KIR3DL1 | Killer cell immunoglobulin like receptor, three Ig domains and long cytoplasmic tail 1 | Immuno-oncologic | P43629 | 0.119 | 0.214 | -0.051 |
| 147 | MCP-3 | C-C Motif Chemokine Ligand 7 | Immuno-oncologic | P80098 | -0.197 | 0.127 | -0.001 |
| 148 | TNFRSF12A | TNF receptor superfamily member 12A | Immuno-oncologic | Q9NP84 | 0.372 | 0.174 | 0.063 |
| 149 | TNFSF14 | TNF superfamily member 14 | Immuno-oncologic | O43557 | 0.024 | 0.187 | -0.051 |
| 150 | TWEAK | TNF Superfamily Member 12 | Immuno-oncologic | O43508 | -0.053 | -0.103 | -0.076 |
| 151 | MMP7 | Matrix Metallopeptidase 7 | Cardiometabolic II | P09237 | 0.486 | 0.020 | 0.211 |
| 152 | EPHB4 | EPH Receptor B4 | Cardiometabolic III | P54760 | 0.442 | -0.043 | 0.129 |
| 153 | SPON1 | Spondin 1 | Cardiometabolic III | Q9HCB6 | 0.509 | 0.058 | 0.394 |
| 154 | CD40 | CD40 Molecule | Immuno-oncologic | P25942 | 0.439 | -0.017 | 0.210 |
| 155 | CSF-1 | Colony Stimulating Factor 1 | Immuno-oncologic | P09603 | 0.432 | 0.037 | 0.190 |
| 156 | ICOSLG | Inducible T Cell Costimulator Ligand | Immuno-oncologic | O75144 | -0.490 | 0.088 | -0.106 |
| 157 | PD-L1 | CD274 Molecule | Immuno-oncologic | Q9NZQ7 | 0.389 | 0.004 | 0.134 |
| 158 | VEGFR-2 | Kinase Insert Domain Receptor | Immuno-oncologic | P35968 | -0.483 | -0.038 | -0.215 |
| 159 | AGRP | Agouti Related Neuropeptide | Cardiometabolic II | O00253 | 0.288 | -0.085 | 0.277 |
| 160 | BMP-6 | Bone Morphogenetic Protein 6 | Cardiometabolic II | P22004 | -0.162 | 0.097 | -0.213 |
| 161 | BOC | Brother Of CDO | Cardiometabolic II | Q9BWV1 | -0.027 | 0.033 | 0.275 |
| 162 | CCL3 | C-C Motif Chemokine Ligand 3 | Cardiometabolic II | P10147 | -0.230 | -0.055 | 0.206 |
| 163 | CTRC | Chymotrypsin C | Cardiometabolic II | Q99895 | -0.138 | 0.086 | 0.164 |
| 164 | CTSL1 | Cathepsin L1 | Cardiometabolic II | P07711 | 0.316 | 0.018 | 0.412 |
| 165 | DKK-1 | Dickkopf WNT Signaling Pathway Inhibitor 1 | Cardiometabolic II | O94907 | -0.171 | 0.016 | -0.107 |
| 166 | PAR-1 | Coagulation Factor II Thrombin Receptor | Cardiometabolic II | P25116 | 0.074 | 0.033 | 0.198 |
| 167 | PGF | Placental Growth Factor | Cardiometabolic II | P49763 | 0.307 | 0.050 | 0.227 |
| 168 | PIGR | Polymeric Immunoglobulin Receptor | Cardiometabolic II | P01833 | -0.276 | 0.071 | 0.306 |
| 169 | PRELP | Proline And Arginine Rich End Leucine Rich Repeat Protein | Cardiometabolic II | P51888 | 0.301 | -0.066 | 0.287 |
| 170 | PRSS27 | Serine Protease 27 | Cardiometabolic II | Q9BQR3 | -0.383 | -0.043 | 0.156 |
| 171 | SPON2 | Spondin 2 | Cardiometabolic II | Q9BUD6 | -0.241 | 0.034 | 0.134 |
| 172 | ALCAM | Activated Leukocyte Cell Adhesion Molecule | Cardiometabolic III | Q13740 | 0.056 | 0.058 | 0.187 |
| 173 | AP-N | Alanyl Aminopeptidase, Membrane | Cardiometabolic III | P15144 | 0.171 | -0.046 | -0.130 |
| 174 | CCL15 | C-C Motif Chemokine Ligand 15 | Cardiometabolic III | Q16663 | 0.115 | 0.039 | 0.368 |
| 175 | DLK-1 | Delta Like Non-Canonical Notch Ligand 1 | Cardiometabolic III | P80370 | 0.068 | -0.053 | 0.301 |
| 176 | FAS | Fas Cell Surface Death Receptor | Cardiometabolic III | P25445 | 0.317 | -0.078 | 0.250 |
| 177 | GP6 | Glycoprotein VI Platelet | Cardiometabolic III | Q9HCN6 | -0.292 | -0.054 | 0.267 |
| 178 | GRN | Granulin Precursor | Cardiometabolic III | P28799 | 0.260 | 0.032 | 0.341 |
| 179 | IL-17RA | Interleukin 17 Receptor A | Cardiometabolic III | Q96F46 | -0.018 | 0.093 | 0.358 |
| 180 | IL-18BP | Interleukin 18 Binding Protein | Cardiometabolic III | O95998 | 0.154 | -0.071 | 0.151 |
| 181 | IL-1RT1 | Interleukin 1 Receptor Type 1 | Cardiometabolic III | P14778 | 0.257 | 0.061 | 0.116 |
| 182 | ITGB2 | Integrin Subunit Beta 2 | Cardiometabolic III | P05107 | 0.145 | 0.097 | -0.336 |
| 183 | LTBR | Lymphotoxin Beta Receptor | Cardiometabolic III | P36941 | 0.383 | -0.026 | 0.201 |
| 184 | MCP-1 | C-C Motif Chemokine Ligand 2 | Cardiometabolic III | P13500 | 0.376 | 0.021 | 0.114 |
| 185 | NOTCH-3 | Notch Receptor 3 | Cardiometabolic III | Q9UM47 | -0.021 | 0.000 | -0.264 |
| 186 | PCSK9 | Proprotein Convertase Subtilisin/Kexin Type 9 | Cardiometabolic III | Q8NBP7 | -0.224 | -0.026 | 0.118 |
| 187 | PDGF-SUBUNIT-A | Platelet Derived Growth Factor Subunit B | Cardiometabolic III | P04085 | -0.286 | -0.008 | -0.341 |
| 188 | SELP | Selectin P | Cardiometabolic III | P16109 | -0.113 | 0.061 | 0.107 |
| 189 | TFPI | Tissue Factor Pathway Inhibitor | Cardiometabolic III | P10646 | 0.260 | -0.063 | 0.168 |
| 190 | TR | Transferrin receptor protein 1 | Cardiometabolic III | P02786 | -0.079 | 0.026 | -0.235 |
| 191 | CCL19 | C-C Motif Chemokine Ligand 19 | Immuno-oncologic | Q99731 | 0.197 | 0.040 | 0.123 |
| 192 | CCL4 | C-C Motif Chemokine Ligand 4 | Immuno-oncologic | P13236 | -0.143 | -0.085 | 0.103 |
| 193 | CD244 | CD244 Molecule | Immuno-oncologic | Q9BZW8 | 0.323 | 0.098 | 0.212 |
| 194 | EGF | Epidermal Growth Factor | Immuno-oncologic | P01133 | -0.297 | -0.024 | 0.113 |
| 195 | FGF2 | Fibroblast Growth Factor 2 | Immuno-oncologic | P09038 | -0.121 | -0.023 | 0.477 |
| 196 | KLRD1 | Killer Cell Lectin Like Receptor D1 | Immuno-oncologic | Q13241 | 0.278 | -0.096 | 0.145 |
| 197 | LAP-TGF-BETA.1 | Transforming Growth Factor Beta 1 | Immuno-oncologic | P01137 | -0.065 | 0.033 | -0.285 |
| 198 | MCP-4 | C-C Motif Chemokine Ligand 13 | Immuno-oncologic | Q99616 | -0.138 | -0.060 | -0.277 |
| 199 | TNF | Tumor Necrosis Factor | Immuno-oncologic | P01375 | -0.119 | -0.068 | 0.106 |
| 200 | VEGFA | Vascular Endothelial Growth Factor A | Immuno-oncologic | P15692 | -0.103 | -0.094 | 0.177 |
| 201 | ADAM-TS13 | ADAM Metallopeptidase With Thrombospondin Type 1 Motif 13 | Cardiometabolic II | Q76LX8 | -0.443 | 0.178 | -0.201 |
| 202 | CEACAM8 | CEA Cell Adhesion Molecule 8 | Cardiometabolic II | P31997 | 0.424 | 0.151 | 0.204 |
| 203 | FS | Follistatin | Cardiometabolic II | P19883 | 0.401 | 0.253 | 0.224 |
| 204 | IL18 | Interleukin 18 | Cardiometabolic II | Q14116 | 0.493 | 0.383 | 0.350 |
| 205 | TIE2 | TEK Receptor Tyrosine Kinase | Cardiometabolic II | Q02763 | -0.440 | 0.128 | -0.159 |
| 206 | TNFRSF13B | TNF Receptor Superfamily Member 13B | Cardiometabolic II | O14836 | 0.500 | 0.111 | 0.110 |
| 207 | CHI3L1 | Chitinase 3 Like 1 | Cardiometabolic III | P36222 | 0.507 | 0.287 | 0.121 |
| 208 | CPA1 | Carboxypeptidase A1 | Cardiometabolic III | P15085 | 0.469 | 0.330 | 0.213 |
| 209 | UPA | Plasminogen Activator, Urokinase | Cardiometabolic III | P00749 | 0.499 | 0.141 | 0.283 |
| 210 | CCL20 | C-C motif chemokine ligand 20 | Immuno-oncologic | P78556 | 0.410 | 0.164 | 0.276 |
| 211 | AMBP | Alpha-1-microglobulin/bikunin precursor | Cardiometabolic II | P02760 | -0.050 | -0.105 | 0.387 |
| 212 | CCL17 | C-C motif chemokine ligand 17 | Cardiometabolic II | Q92583 | -0.103 | -0.144 | -0.322 |
| 213 | CD40-L | CD40 Ligand | Cardiometabolic II | P29965 | -0.010 | 0.149 | 0.110 |
| 214 | FGF-21 | Fibroblast Growth Factor 21 | Cardiometabolic II | Q9NSA1 | 0.223 | 0.181 | 0.163 |
| 215 | GIF | Cobalamin Binding Intrinsic Factor | Cardiometabolic II | P27352 | 0.368 | 0.434 | -0.107 |
| 216 | HB-EGF | Heparin Binding EGF Like Growth Factor | Cardiometabolic II | Q99075 | -0.214 | 0.299 | -0.257 |
| 217 | HOSCAR | Osteoclast Associated Ig-Like Receptor | Cardiometabolic II | Q8IYS5 | 0.169 | -0.187 | -0.117 |
| 218 | IL-27 | Interleukin 27 | Cardiometabolic II | Q8NEV9/Q14213 | -0.086 | -0.190 | 0.316 |
| 219 | ITGB1BP2 | Integrin subunit beta 1 binding protein 2 | Cardiometabolic II | Q9UKP3 | 0.164 | 0.103 | 0.217 |
| 220 | KIM1 | Hepatitis A Virus Cellular Receptor 1 | Cardiometabolic II | Q96D42 | 0.220 | -0.170 | -0.129 |
| 221 | LEP | Leptin | Cardiometabolic II | P41159 | 0.147 | 0.307 | -0.101 |
| 222 | MERTK | MER proto-oncogene, tyrosine kinase | Cardiometabolic II | Q12866 | -0.204 | -0.294 | 0.101 |
| 223 | PDGF-SUBUNIT-B | Platelet Derived Growth Factor Subunit A | Cardiometabolic II | P01127 | -0.374 | -0.135 | -0.382 |
| 224 | PRSS8 | Serine Protease 8 | Cardiometabolic II | Q16651 | 0.345 | 0.219 | 0.289 |
| 225 | PSGL-1 | Selectin P Ligand | Cardiometabolic II | Q14242 | -0.199 | 0.145 | 0.281 |
| 226 | REN | Renin | Cardiometabolic II | P00797 | 0.084 | 0.186 | -0.163 |
| 227 | SERPINA12 | Serpin family A member 12 | Cardiometabolic II | Q8IW75 | -0.187 | 0.158 | -0.427 |
| 228 | SLAMF7 | SLAM family member 7 | Cardiometabolic II | Q9NQ25 | 0.083 | 0.374 | 0.204 |
| 229 | SRC | SRC proto-oncogene, non-receptor tyrosine kinase | Cardiometabolic II | P12931 | -0.333 | 0.186 | 0.268 |
| 230 | TM | Thrombomodulin | Cardiometabolic II | P07204 | 0.344 | 0.142 | 0.310 |
| 231 | VEGFD | Vascular endothelial growth factor D | Cardiometabolic II | O43915 | 0.150 | -0.288 | 0.273 |
| 232 | AZU1 | Azurocidin 1 | Cardiometabolic III | P20160 | 0.324 | 0.218 | -0.114 |
| 233 | CCL16 | C-C motif chemokine ligand 16 | Cardiometabolic III | O15467 | -0.067 | -0.154 | 0.183 |
| 234 | CCL24 | C-C motif chemokine ligand 24 | Cardiometabolic III | O00175 | -0.006 | -0.128 | -0.179 |
| 235 | IGFBP-2 | Insulin Like Growth Factor Binding Protein 2 | Cardiometabolic III | P18065 | 0.279 | -0.268 | 0.110 |
| 236 | IL-1RT2 | Interleukin 1 Receptor Type 2 | Cardiometabolic III | P27930 | -0.103 | 0.426 | -0.351 |
| 237 | MMP-2 | Matrix Metallopeptidase 2 | Cardiometabolic III | P08253 | -0.025 | -0.169 | 0.135 |
| 238 | PAI | Serpin Family E Member 1 | Cardiometabolic III | P05121 | -0.343 | 0.356 | -0.240 |
| 239 | PECAM-1 | Platelet And Endothelial Cell Adhesion Molecule 1 | Cardiometabolic III | P16284 | 0.380 | 0.203 | 0.390 |
| 240 | SELE | Selectin E | Cardiometabolic III | P16581 | 0.117 | 0.311 | -0.145 |
| 241 | CD83 | CD83 molecule | Immuno-oncologic | Q01151 | 0.339 | 0.204 | 0.113 |
| 242 | CXCL11 | C-X-C motif chemokine ligand 11 | Immuno-oncologic | O14625 | -0.100 | 0.131 | -0.285 |
| 243 | IFN-GAMMA | Interferon Gamma | Immuno-oncologic | P01579 | -0.116 | 0.244 | 0.455 |
| 244 | IL15 | Interleukin 15 | Immuno-oncologic | P40933 | 0.287 | 0.121 | 0.290 |
| 245 | MCP-2 | C-C Motif Chemokine Ligand 8 | Immuno-oncologic | P80075 | -0.260 | -0.166 | -0.182 |
| 246 | MIC-A-B | MHC Class I Polypeptide-Related Sequence A, B | Immuno-oncologic | Q29983/Q29980 | -0.007 | 0.224 | 0.173 |
