# Supplementary material for: Exploratory study of serum protein biomarkers for sudden cardiac arrest using protein extension assay: A case-control study
Source: PLoS One. 2025 Feb 24;20(2):e0319466. doi: 10.1371/journal.pone.0319466 (PMC11849859; doi:10.1371/journal.pone.0319466)

S2 Fig. Goslim summary for biological process, molecular function, and cellular component for 97 proteins with strong correlation with sudden cardiac arrest, exceeding the cutoff (|Spearman’s correlation coefficient|>0.516)


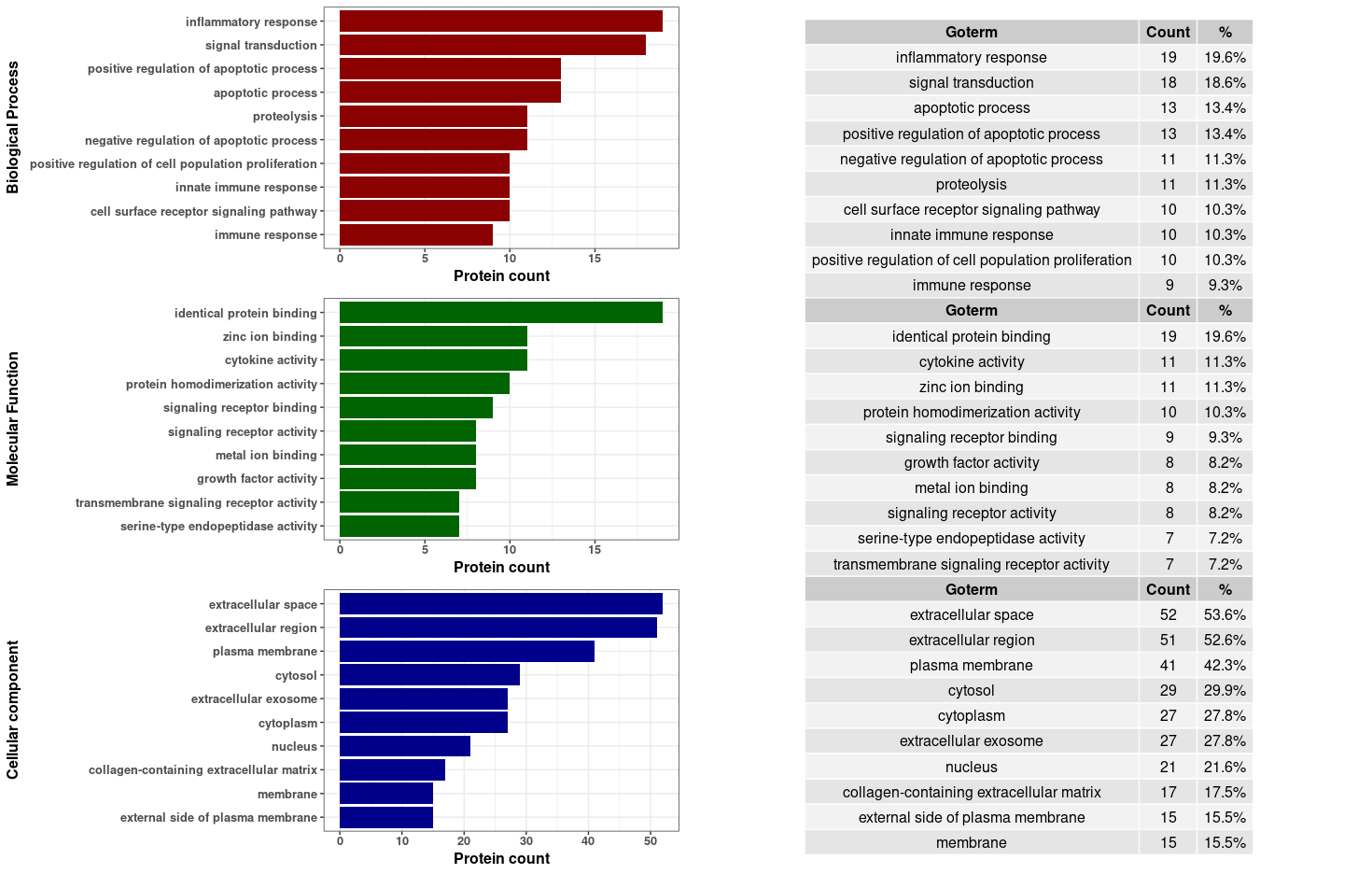

Supplement: S2 Fig — (DOCX) [file pone.0319466.s005.docx]
