# Supplementary material for: Exploratory study of serum protein biomarkers for sudden cardiac arrest using protein extension assay: A case-control study
Source: PLoS One. 2025 Feb 24;20(2):e0319466. doi: 10.1371/journal.pone.0319466 (PMC11849859; doi:10.1371/journal.pone.0319466)

S3 Fig. Distribution of AXL and TIMP-4 in patient groups by center. AXL Receptor Tyrosine Kinase; TIMP Metallopeptidase Inhibitor 4.


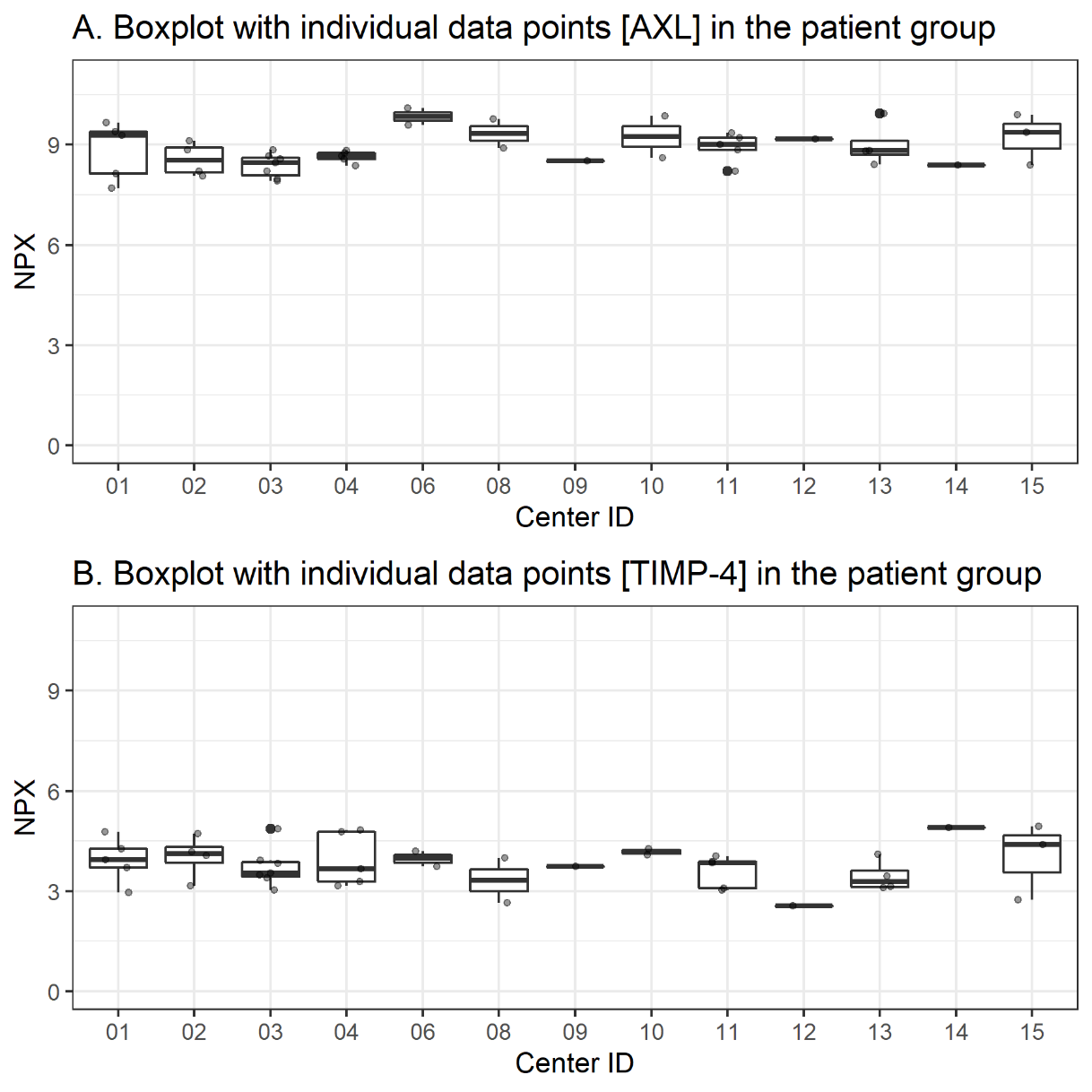

Supplement: S3 Fig — AXL Receptor Tyrosine Kinase; TIMP Metallopeptidase Inhibitor 4. (DOCX) [file pone.0319466.s006.docx]
